# Determination of Emerging Contaminants in Cereals by Gas Chromatography-Tandem Mass Spectrometry

**DOI:** 10.3389/fchem.2020.571668

**Published:** 2020-09-16

**Authors:** Beatriz Albero, José Luis Tadeo, Rosa Ana Pérez

**Affiliations:** Departamento de Medio Ambiente y Agronomía, Instituto Nacional de Investigación y Tecnología Agraria y Alimentaria, Madrid, Spain

**Keywords:** grains, pharmaceuticals, phenols, parabens, ultrasound-assisted extraction, analysis

## Abstract

Cereals are staple foods for human consumption in both developed and developing countries. In order to improve agricultural outputs, resources like reclaimed water for irrigation and biosolids and manure as fertilizers are frequently used, although they may increase the input of contaminants that can potentially be absorbed by crops and enter the food chain. Emerging contaminants (human and veterinary pharmaceuticals, personal care products, surfactants, plasticizers, and industrial additives, among others) are continuously introduced in the environment from a variety sources and these contaminants may enter the food chain through plant uptake. In this study, an analytical method, based on ultrasound-assisted extraction and dispersive solid-phase cleanup, was developed for the determination of emerging contaminants from different classes in four highly consumed cereal grains (wheat, oat, barley, and rice). These analytes were selected considering the results of our previous studies carried out in soil and vegetables and those frequently detected in real samples were chosen. The target compounds selected were bisphenol A (BPA), bisphenol F (BPF), methyl paraben, propyl paraben, linear chain nonylphenol in position 4 (4-n-NP), mixture of ring and chain isomers of NP and six pharmaceutical compounds (allopurinol, mefenamic acid, carbamazepine, paracetamol, diclofenac and ibuprofen). Recoveries ranging from 68 to 119% with relative standard deviations (RSD) <18% were obtained for all the compounds except for allopurinol, with recoveries that ranged from 30 to 66% with RSD ≤ 12% and the limits of detection achieved ranged from 0.03 to 4.9 ng/g. The method was applied to the analysis of 16 cereal samples, ten were purchased in local supermarkets and the rest were collected directly from agricultural fields, five of which were fertilized with organic amendments. Bisphenol A (BPA) was detected in all samples at levels that ranged from 1.6 to 1,742 ng/g. Bisphenol F, a substitute for BPA, was also found in six samples (up to 22 ng/g). Linear 4-n-NP was found in a reduced number of samples but the mixture of NP isomers was found in all the samples, being the mean concentrations in wheat, barley, oat and rice 49, 90, 142, and 184 ng/g, respectively.

## Introduction

Cereals and cereal products are staple foods for human consumption in both developed and developing countries. More than 50% of world daily caloric intake is derived directly from cereal grain consumption, mainly rice, wheat, and corn. Worldwide grain consumption is increasing because of increased population and demand of cereals for feeding livestock. The Food and Agriculture Organization (FAO) forecasts that the consumption of cereals worldwide in 2020/21 will be 2,780 Mt and the global production of cereals by 2025 is expected to reach 2,818 Mt (OECD/FAO, 2016; FAO, 2020). In order to improve agricultural outputs, resources like reclaimed water for irrigation and biosolids and manure as fertilizers, have become frequently used. Although these uses are beneficial, they may increase the input of contaminants into soil that can potentially be taken up by crops and enter the food chain (Wu et al., 2012). The concern on food safety has increased among consumers regarding the presence of contaminants in food that may pose a health risk, particularly for infants and toddlers as they are more vulnerable. In this regard, it has to be taken into account that cereals are among the first foods introduced in the infant diet. The contaminants most commonly determined in cereals or cereal-based food are mycotoxins, pesticides, heavy metals, acrylamide, polycyclic aromatic hydrocarbons, and residues from packaging material (Thielecke and Nugent, 2018; Skendi et al., 2019; Roszko et al., 2020; Yang et al., 2020).

Although the aforementioned contaminants are considered relevant on cereals, there are other pollutants, named as emerging contaminants (ECs), continuously introduced in the environment from a variety sources that may enter the food chain through plant uptake. This group of compounds comprise human and veterinary pharmaceuticals, personal care products, surfactants, plasticizers, and various industrial additives, among others, being the exposure to these substances linked to adverse effects on human health and animals. Their occurrence in different environmental compartments, such as biosolids, effluents from wastewater treatment plants (WWTPs), soil and vegetables is well-documented (Martín-Pozo et al., 2019; Snow et al., 2019). In contrast, studies on ECs levels in cereals are still scarce in the available literature and were mainly focused in the determination of a reduced number of bisphenols and parabens to evaluate the exposure to these compounds through the diet in Sweden, China, Canada, or the United States (Cao et al., 2011; Gyllenhammar et al., 2012; Liao and Kannan, 2013, 2014; Liao et al., 2013a,b). Very recently, a method was developed for the determination of phenols, parabens, and triclosan together with pesticides in cereal-based food (Azzouz et al., 2020). In addition, a multiresidue method was developed in our laboratory focused in the determination of different classes of antibiotics in cereals, being enrofloxacin detected in a rice sample (Albero et al., 2019). In the present work, several ECs were selected considering the results of our previous studies carried out to determine ECs, among other contaminants, in soil and vegetables and those analytes frequently detected in real samples were chosen (Pérez et al., 2012, 2017; Aznar et al., 2014, 2017; Albero et al., 2017). The target compounds selected were two bisphenols (bisphenol A—BPA and bisphenol F—BPF), two parabens (methyl paraben—MeP and propyl paraben—PrP), linear chain nonylphenol in position 4 (4-n-NP), mixture of ring and chain isomers of NP and six pharmaceutical compounds (allopurinol, mefenamic acid, carbamazepine, paracetamol, diclofenac, and ibuprofen).

The different physicochemical properties of ECs and the complexity of the matrix hinders the multiresidue analysis of these compounds in cereals (Albero et al., 2019). In previous studies, the extraction of ECs from cereal grains or cereal-based food was carried out by shaking (Cao et al., 2011; Liao and Kannan, 2013, 2014; Liao et al., 2013a,b), ultrasound assisted extraction (UAE) (Niu et al., 2012; Picó et al., 2019; Azzouz et al., 2020) or pressurized liquid extraction (PLE) with organic solvents (Carabias-Martínez et al., 2006) or hot water (Cortés et al., 2013). After extraction, the clean-up and preconcentration of extracts were performed by solid-phase extraction (SPE), except in the method employing hot water as extraction solvent that applied hollow fiber liquid-phase microextraction (HF-LPME) (Cortés et al., 2013). The determination of ECs in cereals have been usually done by gas or liquid chromatography coupled to tandem mass spectrometry (GC-MS/MS or LC-MS/MS, respectively), being gas chromatography, a lower cost technique offering a good sensitivity, although some compounds need derivatization before their determination. There is still a lack of simple analytical methods for the determination of ECs in crops due to the low concentrations expected, the complexity of the matrices and the characteristics of the compounds. Therefore, the main objective of this study was to develop a selective, sensitive, and efficient analytical method for quantitative determination of different types of ECs in four highly consumed cereals (wheat, barley, oat, and rice) by GC-MS/MS and to provide data regarding their presence in staple food.

## Materials and Methods

### Chemicals and Reagents

Standards of 4-n-NP, BPA, BPF, MeP, PrP, ibuprofen, mefenamic acid, carbamazepine, diclofenac, allopurinol and paracetamol (all purity > 97%), NP technical grade (mixture of ring and chain isomers), N-(tert-butyldimethylsilyl)-N-methyl-trifluoroacetamide (MTBSTFA, purity ≥ 95%) with 1% tert-butyldimethylchlorosilane (TBDMCS) were acquired from Sigma-Aldrich (St. Louis, MO, USA). Ultrahigh purity water was obtained from a MilliQ water purification system (Millipore, Spain).

Acetonitrile (ACN), ethyl acetate (EtAc), and methanol (MeOH), for GC residue analysis, were supplied by Aldrich (Steinheim, Germany). Formic acid (purity ≥ 98%) was acquired from Acros Organics (Geel, Belgium). Ammonium hydroxide (NH_4_OH, ≥32%) and primary secondary amine (PSA) bulk adsorbent were purchased from Scharlab (Barcelona, Spain).

Individual stock solutions (1 mg/mL) of each compound were prepared in ACN, except for diclofenac and paracetamol that were prepared in ACN:MeOH (90:10 v/v), allopurinol in dimethyl sulfoxide and mefenamic acid in EtAc. A working mixture solution at 1 μg/mL was prepared in ACN (except for carbamazepine and diclofenac that were at 4 μg/mL due to their low MS response). All the standard solutions were stored in amber vials at −20°C prior to use.

### Cereal Samples

Barley (B), rice (R), wheat (W), and oat (O) were the cereals chosen for this study. Six samples (two barley, two oat and two wheat samples) were taken directly from agricultural fields located in Madrid (Spain). Four of these samples were from fields amended annually for 3 consecutive years with dewatered (B1, O1) or thermally dried sewage sludge (W2), or with pruning waste composted with sewage sludge (O2), whereas one barley sample (B2) was from a field fertilized once with composted poultry manure and one wheat sample (W1) was from a field with mineral fertilization, which was used in the development of the analytical method. The other ten samples were purchased in grocery stores: two wheat (W3 and W4), two barley (B3 and B4), two oat (O3 and O4), and four rice samples (R1–R4). The grains were separated from chaff manually employing a metallic sieve that was thoroughly cleaned and rinsed with acetone to avoid cross contamination. Samples were maintained in glass jars at −20°C until ground using a laboratory mill.

### Sample Preparation

A 2 g sample of ground cereal grains was placed into a 20 mL glass column with a cellulose paper filter of 2 cm diameter at the bottom and closed with a one-way stopcock. Then, 7 mL of AcEt:MeOH (90:10 v/v) containing 3% of NH_4_OH were added to the column that was sonicated during 15 min in an ultrasonic water bath at room temperature. The extract was subsequently collected in a conical tube with a multiport vacuum manifold and evaporated to dryness. A second extraction of the sample with 7 mL of AcEt:MeOH (90:10 v/v) containing 3% of formic acid was performed. This extract was collected and evaporated to dryness in the same tube of the previous extraction step. The residue was reconstituted to 1 mL with ACN, transferred to a 2 mL-Eppendorf tube and subjected to dispersive solid-phase extraction (dSPE) with PSA (50 mg) vortexing during 2 min and centrifuging 10 min (6,000 rpm, 20°C). An aliquot of the extract was transferred to a 2 mL amber vial for its GC-MS/MS analysis.

In the recovery assays, samples were spiked and stored at 4°C for 24 h before carrying out the extraction procedure.

### Chromatographic Analysis

GC-MS/MS analysis of 11 ECs (NP isomers not included) was performed on an Agilent 7890A (Waldbronn, Germany) gas chromatograph coupled to an Agilent 7000 triple quadrupole mass spectrometer. Separations were carried out using an Agilent HP-5MS ultra inert capillary column (30 m × 0.25 mm i.d. and 0.25 μm film thickness), from Agilent (USA). Helium (purity 99.995%) was used as carrier gas at a flow-rate of 1 mL/min. Two-layer sandwich injections were carried out with the injector port at 300°C into an ultra-inert fritted liner, drawing 1 μl of sample and 0.5 μl of MTBSTFA:TBDMCS (99:1, v/v) in pulsed splitless mode (pulsed pressure 45 psi for 1.5 min and flow rate of 60 mL/min) with the purge valve activated 1.5 min after sample injection. The column temperature was initially set at 80°C (kept for 0.5 min) and increased at 10°C/min to 180°C, then it was programmed at 20°C/min to 280°C (held 8 min). The total analysis time was 23.5 min and the run was carried out with a solvent delay of 10 min.

Firstly, retention times and mass spectra of all analytes were acquired in the full scan mode (mass range from 50 to 500 m/z, scan time of 150 ms and ion source temperature of 230°C). The mass spectrometer was operated in electron impact ionization mode at 70 eV with the electron multiplier set to a gain factor of 4. Precursor ions were chosen taking into account a high ion m/z and abundance. The product ion spectra were obtained by the dissociation of the precursor ions at collision energies ranging from 5 to 50 eV. Multiple reaction monitoring (MRM) was employed for quantitative analysis, using one quantifier and one qualifier transitions to identify each target analyte. Table 1 lists the analytes studied along with their retention times and mass spectrometric parameters. For positive confirmation, quantifier-qualifier ratios must range within 20% uncertainty and retention time must be within ± 0.2 min of the expected time. The quantification of the studied compounds was accomplished by matrix-matched calibration.

**Table 1:** Mass spectrometry parameters and retention times (t_R_) of the 11 ECs for the GC-MS/MS method.

**Compound**	**t_**R**_**	Quantifier transition (CE)^*^	**Qualifier transition (CE)**
MeP	12.46	209 > 177 (10)	209 > 91 (20)
Ibuprofen	13.27	263 > 75 (15)	263 > 161 (20)
PrP	13.67	237 > 151 (10)	237 > 195 (5)
Allopurinol	14.50	307 > 193 (25)	307 > 166 (35)
Paracetamol	14.73	322 > 248 (20)	322 > 150 (35)
4-n-NP	15.06	277 > 165 (10)	277 > 91 (20)
Mefenamic acid	16.52	298 > 224 (20)	224 > 180 (35)
Carbamazepine	16.94	352 > 75 (30)	193 > 167 (25)
Diclofenac	17.22	214 > 179 (30)	214 > 151 (30)
BPF	17.87	179 > 73 (20)	428 > 179 (10)
BPA	18.24	442 > 441 (5)	442 > 73 (20)

* *CE, collision energy (eV)*.

The chromatographic analysis of the mixture of NP isomers (NPs) was based on a method for the analysis of NPs in river water that did not require derivatization (Shimadzu, 2014) that was modified by programming the GC oven to reach 300°C at a faster rate. The eight product ions from four precursors reported by Shimadzu (2014) were tested with collision energies that ranged from 5 to 50 eV. Finally, two transitions were selected, 135 > 107 as quantifier and 135 > 95 as qualifier, both with 15 eV of CE. These transitions are representative of the following NP isomers: 4-(2,4-dimethylheptane-2-yl)-phenol (NP1), 4-(3,6-dimethylheptane-3-yl)-phenol (NP2), 4-(3,5-dimethylheptane-2-yl)-phenol (NP3), 4-(3-ethyl-2-methylhexane-2-yl)-phenol (NP4) and 4-(2,3-dimethylheptane-2-yl)-phenol (NP5). The injection of 2 μL was carried out in pulsed splitless mode (in the same conditions mentioned above) with the injector port at 250°C. The column temperature was kept at 50°C for 1 min and increased at 15°C/min to 300°C. The total analysis time was 17.7 min and the run was carried out with a solvent delay of 8 min.

### Quality Assurance/Quality Control

The quality assurance and quality control criteria used for this method included the analysis of reagent blanks. BPA, MeP, and PrP were detected in some reagent blanks, at levels lower than 4 ng/mL (equivalent to 2 ng/g). One reagent blank was run with each set of three cereal samples to control potential contamination from the preparative steps and to demonstrate laboratory background levels. The concentration of the studied compounds obtained in the analyzed reagent blank samples were subtracted from the concentration levels determined in the cereal samples. In order to avoid memory effects, the liner in the injection port was changed frequently and the injection of neat EtAc was done after three sample injections. In order to minimize BPA contamination, glassware was employed that was thoroughly rinsed with acetone before use. The developed method was validated in terms of linearity, intra-day precision, within-lab reproducibility, accuracy, and detection and quantification limits.

### Statistical Analysis

Data analyses were performed using the statistical package Statgraphics Plus, release 5.0 (Manugistics, Maryland, USA).

## Results and Discussions

### Gas Chromatographic Determination

The ECs studied possess functional groups with active hydrogen atoms so, in order to improve their thermal stability, volatility, and GC behavior, a chemical derivatization of these groups was required before their analysis. Silylation with MTBSTFA:TBDMCS (99:1, v/v) to form t-butyldimethylsilyl derivatives was an effective approach for their determination in aquatic plants (Aznar et al., 2017). The reaction was carried out mixing 100 μL of the extract with 50 μL of derivatizing reagent and keeping the vial at 70°C for 60 min. This off-line procedure was compared with an on-line derivatization carried out in the injection port employing the same reagent:sample ratio. For the on-line reaction, a two-layer injection drawing 1 μL of sample and 0.5 μL of the silylation reagent was performed. The MS responses observed were very similar with both derivatization procedures.

With the on-line approach, the amount of derivatization reagents is greatly reduced and the derivatization time is shortened while the decomposition of the derivatives is avoided or at least reduced. Moreover, injection port derivatization requires less manipulation of toxic reagents compared to manual derivatization. These advantages imply a lower cost of the analysis. In a previous study, the determination of BPA in cereals by GC-MS was carried out employing acetic anhydride in a K_2_CO_3_ solution to form the di-ester derivative of the target analyte that was then extracted with isooctane followed by methyl tert-butyl ether (Cao et al., 2011). This method involved several steps before the chromatographic analysis making the procedure tedious and time-consuming.

The MS response of target analytes may be affected by the presence of matrix components; therefore, the matrix effect was evaluated preparing a set of five standard solutions in the range from 2 to 500 ng/mL in ACN (for diclofenac and carbamazepine the concentrations ranged from 8 to 2,000 ng/mL) and another set spiking blank cereal extracts in the same concentration range. The slopes obtained by plotting concentration at five levels against peak area, following linear regression analysis, were compared. In general, the compounds were slightly affected by the presence of matrix components, with slope ratio ranging from 0.8 to 1.2. Although an increase of the response due to matrix effects is frequently observed in gas chromatographic analyses, in this case, a significant decrease of the MS responses was observed in three analytes (paracetamol, mefenamic acid, and carbamazepine) with slope ratios <0.3, probably due to the presence of coextractives that hindered the derivatization process. Matrix-matched standards were employed to counteract the matrix effects observed.

### Extraction Procedure

The selective extraction of analytes from complex matrices, such as cereals, is a very complicated task, because these matrices contain a large variety of compounds that may hinder their analysis. The optimization of the extraction procedure was carried out with the wheat sample W1 because it was collected from an agricultural field that had not been treated with organic amendments, only with mineral fertilization. In first place, UAE of 2 g of ground wheat grains was performed with EtAc at two pH conditions, using EtAc containing 3% NH_4_OH (7 mL) in a first extraction step followed by two additional UAE steps with EtAc containing 3% formic acid (2 × 5 mL). These starting conditions were selected based on our previous experience in the analysis of ECs in environmental matrices, because the extraction with organic solvent in acidic conditions (ACN or EtAc) enhanced the recovery of acidic compounds, such as mefenamic acid, and a previous extraction with the organic solvent containing NH_4_OH improved the extraction of other pharmaceutical compounds (Aznar et al., 2014, 2017; Albero et al., 2017). The extracts showed turbidity and had to be filtered before their chromatographic analysis. In general, good recoveries (>76%) were obtained for all the target analytes (except for allopurinol), although some of these results showed a high variability. Thus, a cleanup step by dSPE with PSA was assayed to reduce the coextraction of interferences that could cause the variability of these results. With this procedure, cleaner extracts were achieved with similar recoveries of the target compounds, so dSPE was selected as cleanup step for the following assays (data not shown).

**Figure 1:**
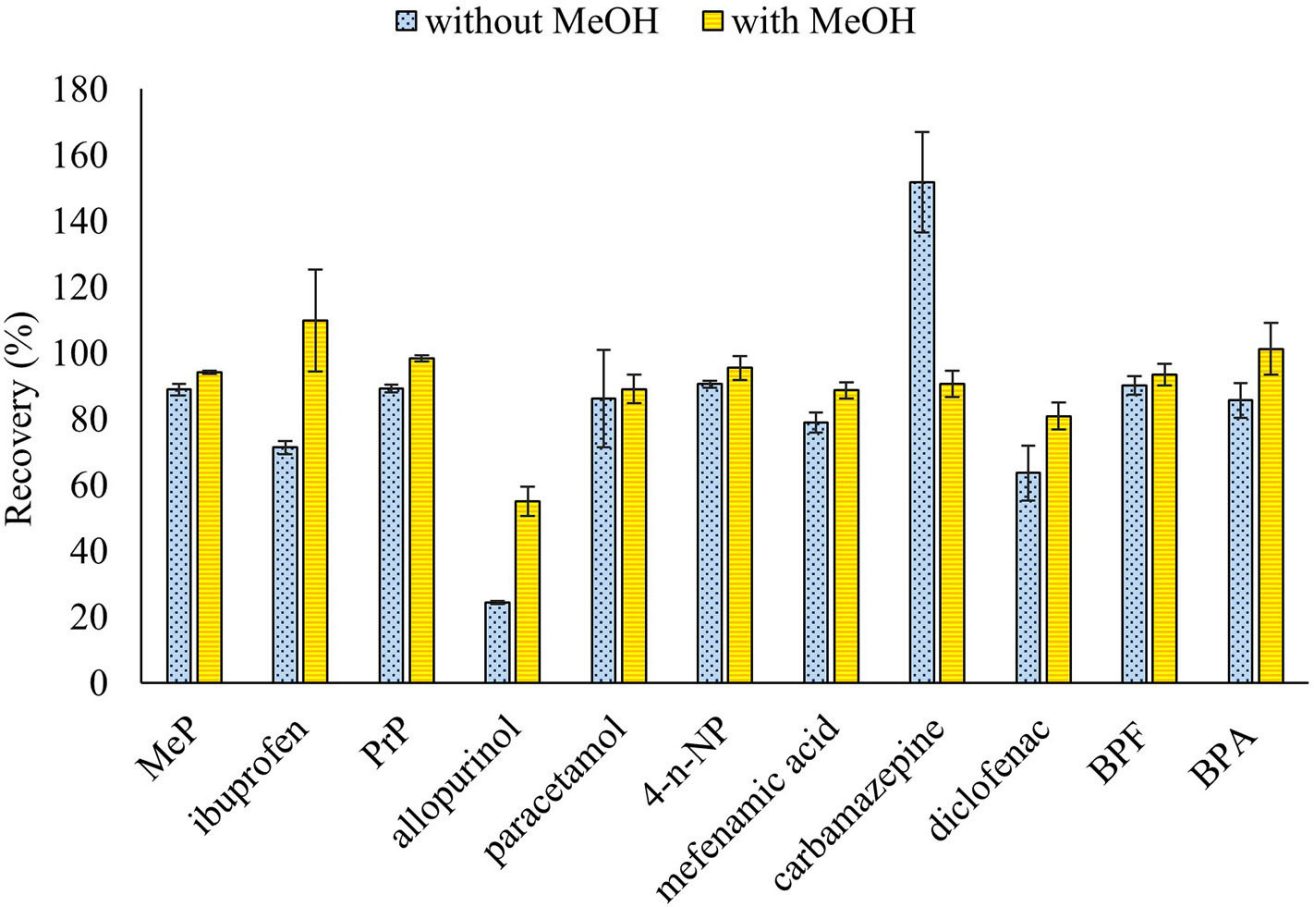
Effect of MeOH in the extraction mixture on the recovery of ECs from ground wheat grains spiked at 150 ng/g. Three UAE cycles, the first with EtAc:MeOH (90:10, v/v) containing 3% of NH_4_OH (7 mL) and the two others with EtAc:MeOH (90:10, v/v) containing 3% of formic acid (5 mL), and the comparison using the mixture without MeOH.

**Figure 2:**
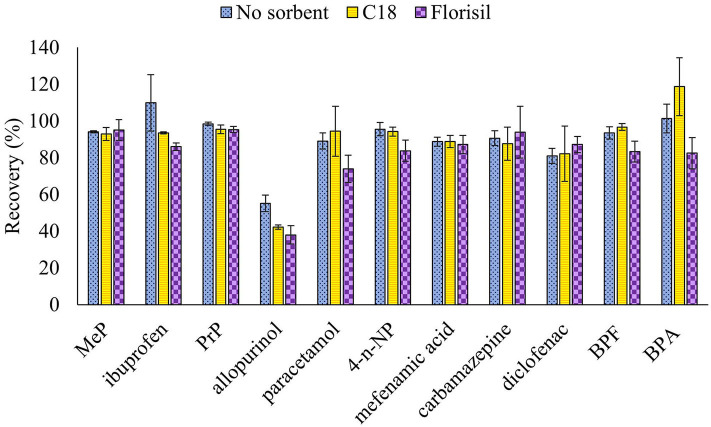
Effect of adding 1 g of sorbent (C18 or Florisil) in the column on the recovery of ECs from ground wheat grains spiked at 150 ng/g. The extraction was carried out with three 15 min sonication cycles.

**Figure 3:**
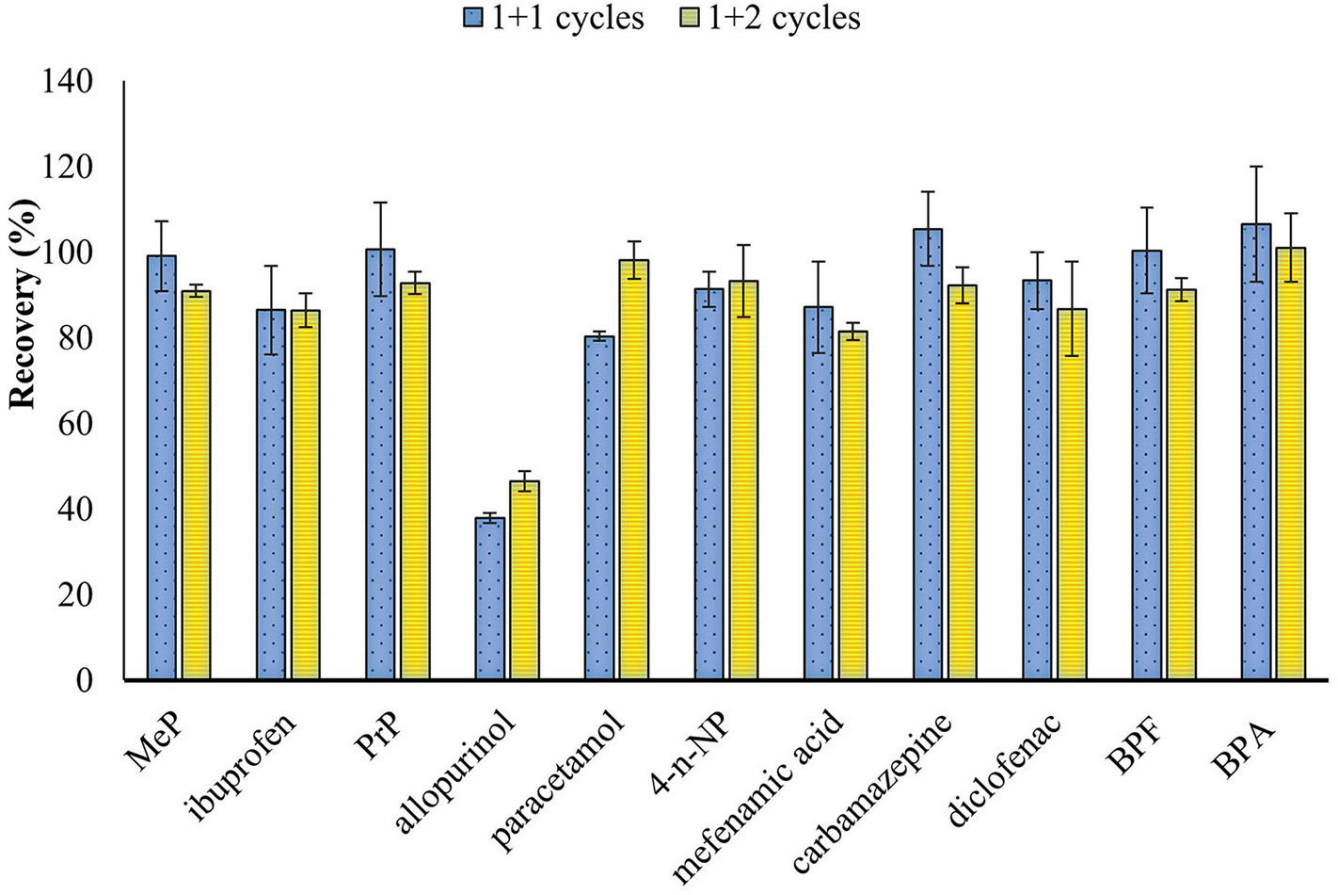
Effect of the sonication cycles on the recovery of ECs from ground wheat grains spiked at 150 ng/g. First cycle with AcEt:MeOH (90:10, v/v) containing 3% of NH_4_OH (7 mL) and the other with AcEt:MeOH (90:10, v/v) containing 3% of formic acid (7 mL for one cycle and 5 mL for two cycles).

The effect of the solvent polarity and the use of sorbents to improve the extraction yields and the cleanup of the samples in the UAE process were evaluated. Thus, an increase of the polarity of the extraction solvent by using a 10% of MeOH in the extraction solutions employed in the previous assay was assessed and better results were obtained in presence of MeOH (Figure 1). In addition, the effect of adding C18 or Florisil (1 g) at the bottom of the column, before placing the sample, to eliminate interferences was evaluated with the basic and acidic extraction solutions containing 10% MeOH, and results were compared with the extraction performed without sorbents. Results showed that similar recoveries were obtained when no sorbent or C18 were used, but a higher variability in the results was observed for some analytes when C18 was used and a decrease in the recovery of some of the evaluated compounds was obtained with Florisil (Figure 2). According to these results, the use of sorbents for a simultaneous extraction and cleanup of samples did not provide a significant improvement of the results, so subsequent assays were done without the addition of sorbents to the column. Finally, in order to shorten the procedure and reduce the consumption of solvents the extraction with two sonication cycles, with AcEt:MeOH (90:10, v/v) containing 3% of NH_4_OH (7 mL) and AcEt:MeOH (90:10, v/v) containing 3% of formic acid (7 mL) was tested and compared to the extraction carried out with three sonication cycles (the first cycle with 7 mL of the basic extraction solution and then the other two cycles with 5 mL of the acidic extraction solution (Figure 3). The results showed that, in general, three sonication cycles did not improve the extraction yields. Therefore, two sonication cycles, followed by a cleanup with PSA by dSPE, were selected for this study.

### Method Validation

After the optimization of the method using wheat grains, the method performance was evaluated for all the cereals selected in this study in terms of linearity, accuracy, repeatability, and limits of detection and quantification for the analysis of the mixture of 11 ECs in a first step and subsequently for the NPs that are discussed afterwards.

The linearity of the method was evaluated injecting a set of five solutions prepared spiking blank grain extracts at a concentration range from 1 to 250 ng/g for the studied compounds (4–1,000 ng/g for carbamazepine and diclofenac). Correlation coefficients were ≥0.99 for all the compounds in the different cereal extracts.

The accuracy of the method was evaluated performing the recovery of target analytes from cereal grains. Samples were spiked with 100 μL of the corresponding standard mixture of the ECs and kept at 4°C until the next day. The recovery was carried out at two spiking levels, 50 and 5 ng/g (except for carbamazepine and diclofenac that were spiked at 200 and 20 ng/g). The recovery at 150 ng/g (600 ng/g for diclofenac and carbamazepine) carried out with wheat samples during the optimization of the extraction method is also included in Table 2. As shown in Table 2, recoveries ranging from 68 to 119% with relative standard deviations (RSD) <18% were obtained for all the compounds except for allopurinol, with recoveries ranging from 30 to 66% with RSD ≤ 12%. Our results were of the same order than those reported by other authors for NP, BPA, BPF, MeP, PrP, diclofenac, and carbamazepine in cereal products (see Table 3) or higher than those reported for ibuprofen and paracetamol in barley that were <59% (Picó et al., 2019). Regarding allopurinol, it has not been previously determined in cereals so we cannot compare our results with those of other authors. Most of the works found in the scientific literature have focused on the determination of allopurinol in biological fluids, such as plasma or urine. Liu et al. (2013) reported recoveries <56% for allopurinol in human urine. In the determination of allopurinol in environmental samples, Aznar et al. (2014) obtained very similar results for soil samples.

**Table 2:** Mean recoveries and relative standard deviations (%) obtained for the selected ECs in different cereal samples (*n* = 3).

	**Wheat**			**Oat**		**Barley**		**Rice**	
	**150 ng/g**	**50 ng/g**	**5 ng/g**	**50 ng/g**	**5 ng/g**	**50 ng/g**	**5 ng/g**	**50 ng/g**	**5 ng/g**
MeP	99 (8)	95 (2)	112 (16)	92 (6)	91 (12)	93 (4)	105 (13)	100 (9)	95 (10)
Ibuprofen	86 (10)	90 (13)	104 (18)	88 (9)	90 (7)	78 (6)	96 (10)	98 (5)	104 (10)
PrP	93 (3)	100 (12)	112 (16)	104 (9)	102 (14)	110 (11)	82 (9)	89 (8)	105 (8)
Allopurinol	55 (4)	66 (6)	43 (5)	40 (12)	31 (7)	38 (3)	45 (9)	30 (7)	37 (10)
Paracetamol	98 (4)	78 (12)	72 (4)	86 (11)	81 (12)	92 (8)	68 (7)	87 (16)	86 (11)
4-n-NP	91 (4)	106 (7)	95 (4)	93 (10)	90 (8)	86 (3)	88 (13)	103 (5)	94 (9)
Mefenamic acid	89 (2)	96 (3)	69 (8)	86 (5)	88 (4)	87 (6)	78 (5)	89 (12)	101 (5)
Carbamazepine^*^	91 (4)	99 (6)	105 (14)	98 (8)	92 (6)	91 (6)	96 (10)	104 (3)	88 (10)
Diclofenac^*^	105 (9)	94 (8)	111 (11)	95 (5)	85 (13)	104 (10)	95 (16)	104 (8)	119 (13)
BPF	100 (10)	100 (3)	92 (5)	104 (10)	100 (10)	98 (8)	94 (8)	94 (4)	114 (4)
BPA	101 (8)	119 (8)	110 (11)	93 (8)	117 (13)	88 (7)	86 (7)	96 (5)	89 (10)

* *Recoveries concentration for wheat: 600–200–20 ng/g, for the other cereals: 200–20 ng/g*.

**Table 3:** Determination of ECs in cereal samples.

**Compounds**	**Samples (cereal)**	**Extraction method**	**Cleanup**	**Analysis**	**LOQ in cereal (ng/g)^**a**^**	**Recovery (%)^**a**^**	**Levels found in ng/g^**a**^ (detection frequency)**	**References**
6 Parabens	282 Foodstuffs (39 cereals and cereal products)	Shaking	SPE	LC-MS/MS	0.01	67–109	MeP: nd^c^-57.6 (97%) PrP: nd-7.8 (72%)	Liao et al., 2013a
6 Parabens	267 Foodstuffs (54 cereal and cereal products)	Shaking	SPE	LC-MS/MS	0.01	82–112	MeP: nd-409 (98%) PrP: nd-31.3 (81%)	Liao et al., 2013b
8 Bisphenols	267 Foodstuffs (48 cereals and cereal products)	Shaking	SPE	LC-MS/MS	0.01–0.05	61–109	BPA: nd-2.5 (56%) BPF: nd-2.6 (2.1%)	Liao and Kannan, 2013
8 Bisphenols	289 Foodstuffs (39 cereals and cereal products)	Shaking	SPE	LC-MS/MS	0.01–0.05	79–109	BPA: nd-130 (69%) BPF: nd-1.3 (12.8%)	Liao and Kannan, 2014
24 ECs	12 Cereal-based foodstuffs	UAE	SPE	GC-MS	0.0005–0.004^b^	82–105	NP: 0.07–0.24 (46%) BPA: 0.023–0.62 (92%) MeP: 0.02–0.45 (54%)	Azzouz et al., 2020
40 ECs	7 Crops (barley)	UAE	SPE	LC-QqTOF-MS	<25	48–94	nd	Picó et al., 2019
4 NSAIDs	Soybean and wheat	PHWE	HF-LPME	LC-MS	1.4^b^		Nd	Cortés et al., 2013
BPA, NP, OP	Cereals (rice, maize, wheat)	UAE	On-line SPE	LC-MS/MS	0.5–1.25	82–116	NP: 9–1,684 (100%) BPA: 1–4 (10–25%)	Niu et al., 2012
7 EDCs	Corn cereals	PLE	SPE	LC-MS	12–43^b^	81–104	nd	Carabias-Martínez et al., 2006
BPA	154 Food composite (rice, wheat, bran, rye)	Shaking	SPE	GC-MS	0.38–1		0.4–1.7	Cao et al., 2011
12 ECs	Wheat, barley, oat, rice grains	UAE	dSPE	GC-MS/MS	0.1–16.2	30–119	MeP: 1–10 (69%) Ibuprofen: 0.5–5 (25%) PrP: 4–8 (12%) 4-n-NP: 0.5–9 (37%) BPA: 2–1,740 (100%) BPF: 12–22 (37%) NPs: 13–484 (100%)	Present work

a *Only for compounds in common with those studied in the present work*.

b *LOD in ng/g*.

c *nd, not detected (< LOD)*.

*ECs, Emerging contaminants; HF-LPME, hollow fiber liquid phase microextraction; NSAIDs, non-steroid anti-inflammatory drugs; PHWE, pressurized hot water extraction; PLE, pressurized liquid extraction; SPE, solid-phase extraction; UAE, Ultrasound-assisted extraction*.

The intra-day precision, or repeatability, was determined by analyzing on the same day seven replicates of each cereal studied that was spiked at 5 ng/g. The intra-day precision, expressed as RSD, was <15% for all compounds in the different cereals. The within-lab reproducibility was evaluated by determining on 3 different days four replicates of each matrix at the lowest spiking levels. The within-lab reproducibility, expressed as RSD, was <23%.

The limits of detection (LODs) and quantification (LOQs) of the developed method were calculated after the analysis of seven replicates of each of the four matrices assayed spiked at the lowest recovery level (5 ng/g) as the minimum amount of analyte detectable with a signal-to-noise ratio of 3 and 10, respectively. As shown in Table 4, the LOQ values for the mixture of 11 ECs ranged from 0.1 to 6.8 ng/g and the LODs from 0.03 to 2.0 ng/g. The limits obtained in the developed method were lower than those reported for pharmaceuticals in cereal products by Cortés et al. (2013) and Picó et al. (2019) (see Table 3). Concerning BPA, our limits are higher than those reported by Liao and Kannan (2013, 2014) and Azzouz et al. (2020) but lower than those included in the studies by Niu et al. (2012) and Carabias-Martínez et al. (2006).

**Table 4:** Limits of detection (LODs, ng/g), quantification (LOQ, ng/g) achieved for the cereals studied (*n* = 7).

**Compound**	**Wheat**		**Oat**		**Barley**		**Rice**	
	**LOD**	**LOQ**	**LOD**	**LOQ**	**LOD**	**LOQ**	**LOD**	**LOQ**
MeP	0.2	0.8	0.1	0.5	0.07	0.2	0.1	0.4
Ibuprofen	0.1	0.4	0.1	0.4	0.1	0.4	0.07	0.2
PrP	0.3	0.9	0.2	0.7	0.4	1.4	0.5	1.8
Allopurinol	0.2	0.6	0.2	0.6	0.05	0.1	0.3	0.9
Paracetamol	0.7	2.4	0.4	1.2	0.2	0.5	0.8	2.8
4-n-NP	0.09	0.3	0.09	0.3	0.06	0.2	0.09	0.3
Mefenamic acid	0.3	0.9	0.1	0.3	0.2	0.6	0.8	2.6
Carbamazepine	2.0	6.8	0.3	0.9	1.3	4.4	0.7	2.5
Diclofenac	0.6	2.1	0.7	2.4	0.5	1.6	0.7	2.3
BPF	0.08	0.3	0.05	0.2	0.05	0.2	0.2	0.8
BPA	0.4	1.2	0.03	0.1	0.04	0.1	0.2	0.7
NP isomers	4.9	16.2	0.7	2.4	2.4	7.8	3.2	10.7

Regarding the analysis of the NPs, the extraction procedure developed for the 11 ECs studied was tested carrying out recovery studies with the four cereals spiked with a mixture of NPs at a concentration of 50 ng/g. The developed method showed good recoveries and reproducibility for the NPs in all the cereal matrices (≥92%, with RSD <9%). The LOD and LOQ for NPs were calculated after the analysis of eight replicates of extract from wheat, barley, oat and rice at the level of 50 ng/g. As shown in Table 4, the LOQ values ranged from 2.4 to 16.2 ng/g. These values are in the range of those reported by Carabias-Martínez et al. (2006).

### Application to Real Samples

The developed method was applied to the analysis of ECs in 16 samples of cereals that were collected directly from agricultural fields or purchased in grocery stores. Table 5 depicts the concentrations of ECs residues found in the samples analyzed. BPA was detected in all the samples with a high variability in the levels found that ranged from 1.6 to 1,740 ng/g. The highest BPA levels were detected in samples that were collected from fields treated with different organic amendments, 173 to 1,740 ng/g. Due to the low number of samples evaluated and the ubiquitous presence of this compound in cereals, further studies should be done in order to assess the effect of the organic soil treatments in the BPA levels. However, the presence of this compound in all samples indicated that BPA should be one of the target compounds to be analyzed in cereals. BPA was the EC most commonly determined in cereals or cereal products and the levels and frequency of detection were variable (Table 3). In general, lower BPA levels than those found in the present work were reported in previous works. In a survey carried out in nine Chinese cities where 289 food samples were collected, BPA was found in 69% of the 39 cereal or cereal-based food samples analyzed with levels that ranged up to 130 ng/g found in baked bread (Liao and Kannan, 2014). However, when Liao and Kannan (2013) monitored BPA and analogs in food in the United States, the levels of BPA in cereal products did not exceed 2.5 ng/g. Cao et al. (2011) detected low levels of BPA (0.4–1.73 ng/g) in some composite samples of bread and cereals; although they also observed BPA in yeast (8.52 ng/g) and reported that the low levels of background BPA found in bread could be due to the yeast. Very low levels of BPA (from 0.02 to 0.62 ng/g) have been recently reported by Azzouz et al. (2020) in 13 different cereal-based food; thus, BPA was found in all the samples except “sesame regañas.”

**Table 5:** Mean concentration and standard deviation (ng/g) of the ECs detected in cereal samples (*n* = 3).

**Sample**	**MeP**	**Ibuprofen**	**PrP**	**4-n-NP**	**BPA**	**BPF**	**NP isomers^**c**^**
**WHEAT**							
W1^*^	nd^a^	nd	nd	nd	33 ± 7	nd	58 ± 3
W2^*^	2.8 ± 0.2	nd	nd	nd	244 ± 33	nd	53 ± 33
W3**	nq^b^	nd	nd	0.5 ± 0.3	2.6 ± 0.7	nd	nq
W4**	nd	nd	nd	nd	126 ± 7	22 ± 4	37 ± 7
**BARLEY**							
B1^*^	10 ± 2	nd	nd	nd	554 ± 38	nd	85 ± 20
B2^*^	7.9 ± 0.9	nd	nd	9.1 ± 0.2	1740 ± 370	nd	49 ± 20
B3**	nq	nd	nd	0.5 ± 0.1	2.5 ± 0.9	nd	13 ± 3
B4**	nq	0.5 ± 0.1	nd	0.7 ± 0.2	1.6 ± 0.2	nd	215 ± 19
**OAT**							
O1^*^	8 ± 1	2.3 ± 0.8	4.5 ± 0.5	nd	374 ± 78	nd	142 ± 57
O2^*^	1.5 ± 0.1	nd	nd	0.60 ± 0.10	173 ± 26	nd	215 ± 90
O3**	nd	nd	nd	nd	104 ± 39	14.9 ± 0.6	52 ± 8
O4**	1.7 ± 0.2	nq	nd	0.60 ±0.10	2.3 ± 0.1	nq	158 ± 13
**RICE**							
R1**	4.9 ± 0.8	nd	8.0 ± 0.3	nd	42 ± 18	14 ± 1	105 ± 4
R2**	7.7 ± 0.2	nd	nd	nd	15 ± 7	nd	484 ± 31
R3**	nd	nd	nd	nd	65 ± 25	12.5 ± 0.6	108 ± 9
R4**	nd	4.9 ± 1.4	nd	nd	47 ± 7	16 ± 2	39 ± 20

* *Samples collected from fields*.

** *Samples collected from supermarkets*.

a *nd, not detected (< LOD)*.

b *nq, not quantified (< LOQ)*.

c *The sum of NP isomers*.

The other bisphenol studied, BPF, was mainly found in rice samples at lower levels (12.5–22 ng/g) and less frequently than BPA (31%). BPF is one of the main substitutes of BPA in the manufacturing of polycarbonate plastics and epoxy resins. The analysis of soil samples from several Spanish areas showed BPF in all industrial soils and in one agricultural soil sample (Pérez et al., 2017). The levels found in our study are higher than those reported 7 years ago for cereal products, but these higher values could be expected as a consequence of replacing BPA by other analogs (Liao and Kannan, 2013, 2014).

Ibuprofen was the only pharmaceutical compound detected in three cereal samples at levels that ranged from 0.5 to 4.9 ng/g. The presence of this compound in cereal grains has not been previously reported. Nevertheless, the use of wastewater effluents for crop irrigation could result in the uptake of pharmaceuticals by plant roots and their translocation to edible parts, thus entering the food chain (Madikizela et al., 2018). Additionally, an increase of pharmaceutical residues in soil is expected due to the growing demand for alternative irrigation resources and the increasing application of sewage sludge to agricultural fields.

In our study, MeP was detected in 69% of the samples but quantified only in 50% of them with concentration levels that ranged from 1.5 to 10 ng/g. PrP was only found in two samples (oat and rice) at levels up to 8.0 ng/g. Liao et al. evaluated the occurrence of parabens in foodstuff from China and the United States (Liao et al., 2013a,b) and the frequency of detection of MeP and PrP was very similar in both countries, 97–98% for MeP and 72–81% for PrP. Furthermore, the mean concentration of both parabens was also analogous, being in China MeP: 16.6 ng/g and PrP: 1.9 ng/g and in the USA MeP: 14.4 ng/g and PrP: 1.2 ng/g, although the range of concentrations found were higher in products from the United States (see Table 3). Seven parabens were analyzed by Azzouz et al. (2020) and MeP, ethylparaben and isopropropylparaben were detected in 46–54% of the samples at very low levels (0.02–0.45 ng/g) but PrP was not detected in any sample. The lower levels and frequency of detection in our study may be explained by the banning of five parabens in the European Union and the new maximum concentration of PrP and butylparaben allowed in cosmetic products, 0.14% when used individually or together instead of the 0.4% when used individually or 0.8% when used combined that were previously permitted (European Commission, 2014).

Finally, 4-n-NP was detected in six samples although only in one sample the concentration was above 1 ng/g. Recently, Azzouz et al. (2020) reported very low levels of 4-n-NP (from 0.09 to 0.24 ng/g) in 46% of the samples analyzed. The fact that 4-n-NP had a low detecting frequency in our real samples showed that this isomer may not be a good indicator of the presence of NPs in samples and it was necessary the determination of other NP isomers. This hypothesis was mainly based on the fact that NPs have been described as ubiquitous in the environment, and high concentrations have been detected in fatty and non-fatty food (Guenther et al., 2002; Lu et al., 2007). Niu et al. (2012) reported that linear and branched isomers of 4-NP were found in all the cereal samples analyzed (a total of 42 samples of rice, maize and wheat).

The analysis of the 16 samples of cereals showed that NPs were detected in all the samples with levels that ranged from 13 to 484 ng/g. Our levels were in line with those reported by Niu et al. (2012), with mean values of 43.3, 139.4, and 396.8 ng/g for rice, maize, and wheat, respectively (with a concentration range from 9.4 to 1683.6 ng/g). As indicated above, five of the cereal samples came from fields treated with different organic amendments but the NPs content of these samples was in the same range that the levels found in samples purchased from local supermarkets (Table 5). The mean concentration of NPs collected from fields treated with organic amendments was 109 ng/g whereas it was 126 ng/g for the other samples. The mean concentrations in wheat, barley, oat and rice were 49, 90, 142, and 184 ng/g, respectively.

## Conclusions

A selective and efficient analytical method, based on UAE and dSPE cleanup, was developed for the determination of different types of ECs in cereal grains by GC-MS/MS. The method was validated for four highly consumed cereals (wheat, rice, oat, and barley). The developed method showed satisfactory recovery values for most compounds, and LOQs were from 0.1 to 16.2 ng/g.

BPA was found in all the samples, being the highest levels detected in samples collected from fields treated with different organic amendments, therefore further studies should be conducted in order to assess the effect of organic soil amendments on BPA levels in cereals. The greater relative presence of BPF, compared to works carried out a few years ago, could be due to the fact that this bisphenol is a substitute for BPA in industrial processes. An increase of levels of BPF and other ECs, such as some pharmaceutical compounds, in environmental and foodstuffs matrices should be expected due to the growing use of recycled water, and the increasing application of organic amendment to agricultural fields. The results obtained in our study showed that 4-n-NP, which is usually used as an indicator of the presence of NPs in food samples, is not a good target compound and it is important to determine other NP isomers. These NPs were detected in the 100% of the samples analyzed. In summary, the detection of BPA and NPs in all cereal samples evaluated points out the importance of knowing the concentration of these compounds in cereals and to further assess the effect of agricultural practices in their levels.

## Data Availability Statement

The original contributions presented in the study are included in the article, further inquiries can be directed to the corresponding author.

## Author Contributions

All authors listed have made a substantial, direct and intellectual contribution to the work, and approved it for publication.

## Conflict of Interest

The authors declare that the research was conducted in the absence of any commercial or financial relationships that could be construed as a potential conflict of interest.
